# Impact of Proteinuria and Kidney Function Decline on Health Care Costs and Resource Utilization in Adults With IgA Nephropathy in the United States: A Retrospective Analysis

**DOI:** 10.1016/j.xkme.2023.100693

**Published:** 2023-06-25

**Authors:** Edgar V. Lerma, Mark E. Bensink, Kamlesh M. Thakker, Richard Lieblich, Martin Bunke, Andrew Rava, Kaijun Wang, Michael V. Murphy, David Oliveri, Diana T. Amari, David M.W. Cork, Juan Carlos Q. Velez

**Affiliations:** 1 University of Illinois Chicago/Advocate Christ Medical Center, Oak Lawn, IL; 2 Travere Therapeutics, Inc, San Diego, CA; 3 Notting Hill Consulting LLC, Celebration, FL; 4 VJA Consulting, Walnut Creek, CA; 5 CM Bunke Consulting, Mt Pleasant, SC; 6 Genesis Research, Hoboken, NJ; 7 Genesis Research, Newcastle upon Tyne, United Kingdom; 8 Department of Nephrology, Ochsner Health, New Orleans, LA; 9 Ochsner Clinical School, The University of Queensland, Brisbane, QLD, Australia

**Keywords:** Chronic kidney disease, costs, health care resource utilization, immunoglobulin A nephropathy, prevalence, proteinuria

## Abstract

**Rationale & Objective:**

Among patients with IgA nephropathy (IgAN), proteinuria and decline in kidney function may be associated with increased economic burden. This study aimed to provide current information on the epidemiology and economic burden of IgAN in the United States.

**Study Design:**

Retrospective cohort study.

**Setting & Study Population:**

Overall, 9,984 patients in the Optum’s Market Clarity database identified by the presence of at least 2 natural language processing-derived IgAN signs and disease and symptoms terms; 813 with linked claims data included in a health care resource utilization/cost subcohort.

**Predictor:**

High-risk proteinuria (≥1 g/d), chronic kidney disease (CKD) stage.

**Outcomes:**

Standardized prevalence, health care resource utilization, costs.

**Analytical Approach:**

Descriptive statistics for categorical and continuous variables. Direct standardization for prevalence estimation. Generalized linear models for health care resource utilization/costs, reported as per-patient-per-month (PPPM) costs in 2020 US dollars.

**Results:**

The estimated standardized US prevalence of IgAN (2016-2020) was 329.0 per 1,000,000 persons. High-risk proteinuria (≥1 vs <1 g/d) was associated with a higher mean PPPM number of outpatient visits (3.49 vs 1.74; *P* = 0.01) and pharmacy claims (3.79 vs 2.41; *P* = 0.01), contributing to higher mean total costs PPPM ($3,732 vs $1,457; *P* = 0.01). Furthermore, higher CKD stage was also associated with higher health care resource utilization (number of outpatient visits PPPM, number of pharmacy claims PPPM, proportion of patients with inpatient visits and emergency department visits; *P* < 0.001) and mean total cost PPPM (from $2,111 CKD stage 1 to $10,703 CKD stage 5/kidney failure; *P* < 0.001).

**Limitations:**

Generalizability outside of the catchment group for the database, missing data/errors inherent in retrospective database studies, relatively small sample size, use of Optum Market Clarity standardized pricing algorithms, exclusion of out-of-pocket costs.

**Conclusions:**

Health care resource utilization and costs were higher for IgAN patients with high-risk proteinuria and worsening kidney function. Treatments that reduce proteinuria and slow CKD disease progression may reduce the economic burden associated with IgAN.

**Plain-Language Summary:**

Immunoglobulin A nephropathy (IgAN) is a rare kidney disease. Over time, the kidneys may leak protein into the urine (proteinuria). IgAN can lead to kidney failure. Because IgAN is rare, it is hard to know how many people have it. This study used electronic health records to estimate the number of patients with IgAN in the United States, describe the characteristics of patients, and understand their treatments and the costs. The number of patients with IgAN increased between 2016 and 2020. The researchers think this is because doctors learned more about IgAN. Patients with severe disease used more health care resources and had higher costs. The authors believe treatments that slow kidney damage may reduce the cost of treating IgAN.

## Graphical abstract

**Figure:**
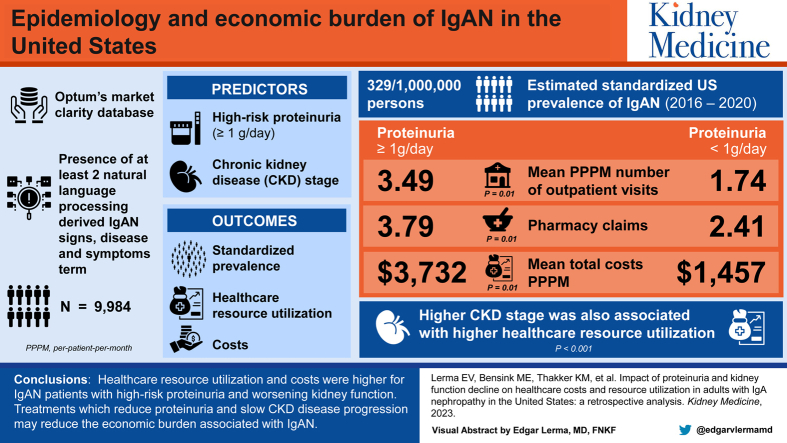

Immunoglobulin A nephropathy (IgAN) is an immune complex-mediated inflammatory disease that, although rare at the population level, is the most common primary glomerular disease in young White adults in the United States.^1^ IgAN is often progressive^2^ and, if not controlled, is a major cause of kidney failure, which impacts patients profoundly because of physical limitations, pain, and fatigue.^3^, ^4^, ^5^ Diagnosis of IgAN is typically made through kidney biopsy.

The current goal of therapy in IgAN, in accordance with the 2021 Kidney Disease: Improving Global Outcomes (KDIGO) guidelines, is to preserve kidney function through management of blood pressure and proteinuria, which is pivotal in slowing progression to kidney failure.^6^ Current guidelines for IgAN recommend initial therapy with either an angiotensin-converting enzyme inhibitor or angiotensin receptor blocker. Maximum conservative therapy should be administered for at least 90 days before the introduction of additional treatments. Corticosteroid therapy is not recommended in patients with estimated glomerular filtration rate (eGFR) <30 mL/min/1.73 m^2^; however, it may be used for patients who remain at high risk for progressive kidney disease despite maximal supportive care whose risk/benefit profile is assessed individually based on a toxicity risk stratification considering advanced age, diabetes, metabolic syndrome, obesity, and latent infection.^6^ The 2021 KDIGO guidelines recognize proteinuria ≥0.75-1 g/d as indicative of a high risk for progression and recommend reduction to <1 g/d as a therapeutic goal.^6^ The guidelines specify that a dynamic assessment of patient risk over time should be performed to guide decisions in treatment choice.^6^ However, there remains a high unmet clinical need due to limited effects of current treatments directed at reducing proteinuria,^7^^,^^8^ difficulty in the long-term stabilization of kidney function,^9^ and significant risk of toxicity associated with corticosteroid therapy.^10^

There are limited reports of analyses using a retrospective database to assess the epidemiology, health care resource utilization, and costs in patients with IgAN in the United States. A recent systematic literature review did not identify any studies reporting the prevalence of IgAN in the United States.^11^ The same publication also identified only 3 studies assessing the economic burden of IgAN, including 2 retrospective database studies (Canada and China) and 1 economic model based in Japan.^11^, ^12^, ^13^, ^14^ Therefore, the overall aim of this study was to provide current information on the epidemiology and economic burden of IgAN in the United States. Specifically, we aimed to estimate the prevalence of IgAN in the US population, describe characteristics of patients with IgAN, and estimate all-cause health care resource utilization and costs for patients with IgAN stratified by proteinuria level (≥1 g/d vs <1 g/d) and chronic kidney disease (CKD) stage.

## Methods

### Study Design and Data Source

This was a descriptive, noninterventional, retrospective cohort study using Optum’s deidentified Market Clarity and proprietary natural language processing (NLP) Data from January 1, 2007 to March 31, 2021. The Optum deidentified Market Clarity Dataset deterministically links electronic health record data from providers across the care continuum with historical, linked administrative claims data, pharmacy claims, physician claims, and facility claims (with clinical information) and is inclusive of medications prescribed and administered. It is fully Health Insurance Portability and Accountability Act (HIPAA)-compliant, statistician-certified, deidentified data. The Optum NLP system was developed using vocabulary from the Unified Medical Language System that includes multiple medical dictionaries such as the Logical Observation Identifiers Names and Codes, the Systemized Nomenclature of Medicine-Clinical Terms, and RxNorm, a listing of generic and branded drugs (among others). NLP concepts are identified and created based on broad topics such as medications, signs, disease and symptoms terms, measurements, observations, etc. The data are harvested from the notes fields within the electronic medical records provided to Optum from over 50 large health care systems throughout the United States. The data used for development of each NLP concept are deidentified, and their accuracy is verified through a series of quality assurance steps before release for use. Each NLP concept included in the data are associated with a unique subject record and a date of observation, allowing longitudinal tracking of concepts over time. As the study is retrospective and observational in nature and uses deidentified patient data, it has been exempted from Institutional Review Board review.

### Study Population

**Figure 1:**
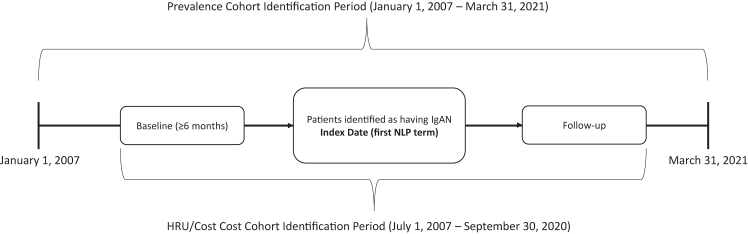
Study design. *Note:* Patient identification period July 1, 2007-September 30, 2020, follow-up ≥6 months for health care resource utilization/cost cohort. Abbreviations: HRU, health care resource utilization; IgAN, immunoglobulin A nephropathy; NLP, natural language processing.

Limited data were available on the presence of a kidney biopsy; therefore, patients in the prevalence cohort included those with at least 2 disease and symptoms term entries with “IgA nephropathy,” “immunoglobulin A nephropathy,” “Berger’s disease,” “Berger’s nephropathy,” “IgA glomerulonephritis,” or “immunoglobulin A glomerulonephritis” within 180 days and at least 30 days apart during the study period (January 1, 2007 and March 31, 2021; Fig 1). Patients with negation terms (eg, “deny,” “failed,” “ignore,” “n/a,” “negative,” “question,” “reject,” “rule out,” “uncertain,” “unspecified”) in relation to the IgAN disease and symptoms term were excluded (Fig S1). The index date was the first IgAN NLP term within the study period.

To be included in the health care resource utilization/cost cohort, patients were required to be included in the prevalence cohort, be at least 18 years old at index date, have at least 6 months of pre-index continuous enrollment (baseline), have at least 6 months of post-index continuous enrollment (follow-up), and have linked Optum Market Clarity claims data. Patients were excluded from the health care resource utilization/cost cohort if they had evidence of a cancer diagnosis at any time during the study period, were pregnant in the pre-index period, or had evidence of COVID-19 at any time during the study period. The identification period for these patients was between July 1, 2007 and September 30, 2020, to account for the baseline and follow-up periods (Fig 1). The index date was the first IgAN NLP term within this identification period.

### Study Outcomes

The standardized prevalence per 1,000,000 US population was estimated for the period of January 01, 2016-December 31, 2020, including per year and across the study period.

Demographics, including age, gender (male, female, unknown), region, ethnicity, race, insurance type, and Charlson Comorbidity Index^15^^,^^16^ were extracted at baseline. Baseline eGFR was calculated without the race modifier, using the 2021 Chronic Kidney Disease Epidemiology Collaboration (CKD-EPI) equation,^17^ and CKD stage was identified based on eGFR values or by diagnosis code. Baseline proteinuria was reported as g/d. If multiple values were available for these data, the value closest to the index date was used. Baseline cardiovascular events were defined as the occurrence of at least one hospital admission with myocardial infarction, ischemic stroke/transient ischemic attack, unstable angina, or congestive heart failure, or at least one inpatient or outpatient revascularization procedure (percutaneous coronary intervention, coronary artery bypass allograft) identified using *International Classification of Diseases, Ninth or Tenth Revision* (ICD-9/ICD-10) diagnosis codes, ICD-9/ICD-10 procedure codes, or Current Procedural Terminology codes.

Health care resource utilization and cost outcomes were reported per-patient-per-month (PPPM) and included inpatient visits, emergency department (ED) visits, outpatient visits, pharmacy claims, and total costs. All costs were adjusted to 2020 US dollars using the Consumer Price Index.

### Statistical Analyses

Baseline demographic and clinical characteristics were analyzed descriptively, including counts and percentages for discrete data and means with standard deviations for continuous data; 95% confidence intervals were reported as applicable.

Standardized annual and across the study prevalence and confidence intervals were estimated using direct methods to standardize to the age, gender, and race/ethnicity distribution of the general US population, using data from the 2021 US Census Bureau.

Proteinuria data were presented as g/d with urinary protein-creatinine ratio values in g/g converted to g/d. Patients with baseline CKD stage 5/kidney failure, defined as eGFR <15 mL/min/1.73 m^2^, a diagnosis code for CKD stage 5, or the presence of a claim associated with dialysis or a kidney transplant during the baseline period, were excluded from the proteinuria-based analyses because proteinuria measurements are likely to be unreliable at advanced stages of CKD.

For categorical health care resource utilization outcomes, comparisons were assessed using the Fisher exact test. For differences in continuous health care resource utilization and cost outcomes by proteinuria level and by CKD stage, a generalized linear regression was conducted to analyze differences in means, and the Jonckheere-Terpstra test was conducted to analyze differences in medians across groups.

## Results

### Baseline Demographics and Clinical Characteristics

The prevalence cohort consisted of 9,984 patients with a mean age of 44.9 years (91.9% aged ≥18 years) and a median (first quartile-third quartile [Q1-Q3]) of 44.0 (22.3-72.0) months of post-index follow-up (Table 1). Fifty-seven percent of patients were male; 74.1% were White. Median (Q1-Q3) proteinuria in the prevalence cohort was 1.2 (0.3-3.3) g/d, and mean (standard deviation) proteinuria was 2.8 (4.7); this discrepancy is likely due to outliers increasing the mean proteinuria estimate. Median (Q1-Q3) eGFR was 56.3 (25.5-93.0) mL/min/1.73 m^2^. Among patients with available eGFR data or a diagnosis code, 61.0% were in CKD stages 3-5 (Table 1). A small percentage of patients experienced a baseline cardiovascular event (1.7%) or a baseline kidney failure event (dialysis or kidney transplant; 1.1%), and median Charlson Comorbidity Index was mild (1.0 [0.0-1.0]).

**Table 1:** Baseline Patient Demographics and Clinical Characteristics Among Patients With IgAN: Prevalence and Health Care Resource Utilization/Cost Cohorts, 2007-2021

Characteristics	Prevalence Cohort (n = 9,984)	Health Care Resource Utilization/Cost Cohort (n = 813)
**Age, y**		
Mean (SD)	44.9 (17.8)	47.4 (13.7)
Median (Q1-Q3)	46.0 (33.0-58.0)	48.0 (38.0-57.0)
**Age, n (%)**		
<18 y	802 (8.0%)	0 (0.0%)
18-45 y	4,106 (41.1%)	350 (43.1%)
46-65 y	3,848 (38.5%)	393 (48.3%)
>65 y	1,224 (12.3%)	70 (8.6%)
Unknown	4 (0.0%)	0 (0.0%)
**Gender, n (%)**		
Female	4,271 (42.8%)	303 (37.3%)
Male	5,707 (57.2%)	510 (62.7%)
Unknown	6 (0.1%)	0 (0.0%)
**Region, n (%)**		
Midwest	4,523 (45.3%)	326 (40.1%)
Northeast	1,670 (16.7%)	208 (25.6%)
Other/unknown	417 (4.2%)	34 (4.2%)
South	1,748 (17.5%)	124 (15.3%)
West	1,626 (16.3%)	121 (14.9%)
**Ethnicity, n (%)**		
Hispanic	815 (8.2%)	57 (7.0%)
Not Hispanic	8,228 (82.4%)	668 (82.2%)
Unknown	941 (9.4%)	88 (10.8%)
**Race, n (%)**		
African American	481 (4.8%)	32 (3.9%)
Asian	958 (9.6%)	82 (10.1%)
White	7,401 (74.1%)	609 (74.9%)
Other/unknown	1,144 (11.5%)	90 (11.1%)
**Insurance type, n (%)**		
Commercial	5,726 (57.4%)	554 (68.1%)
Medicaid	1,372 (13.7%)	92 (11.3%)
Medicare	2,235 (22.4%)	146 (18.0%)
Other payor type	236 (2.4%)	2 (0.2%)
Uninsured	254 (2.5%)	9 (1.1%)
Unknown	161 (1.6%)	10 (1.2%)
**Post-index activity, mo**		
Mean (SD)	50.3 (34.6)	43.4 (27.7)
Median (Q1-Q3)	44.0 (22.3-72.0)	39.7 (21.9-59.8)
**Baseline eGFR, mL/min/1.73 m**^**2**^		
Mean (SD)	62.0 (42.1)	58.1 (34.8)
Median (Q1-Q3)	56.3 (25.5-93.0)	57.2 (29.2-86.6)
**Baseline CKD stage, n (%)**		
Unknown	3,511 (35.2%)	229 (28.2%)
With available data	6,473 (64.8%)	584 (71.8%)
Stage 1: eGFR >90 or CKD diagnosis	1,447 (22.4%)	104 (17.8%)
Stage 2: eGFR 60-89 or CKD diagnosis	1,080 (16.7%)	108 (18.5%)
Stage 3: eGFR 30-59 or CKD diagnosis	1,554 (24.0%)	167 (28.6%)
Stage 4: eGFR 15-29 or CKD diagnosis	826 (12.8%)	77 (13.2%)
Stage 5: eGFR <15 or CKD diagnosis	1,566 (24.2%)	128 (21.9%)
**Baseline KF event**^a^**(dialysis or kidney transplant), n (%)**	112 (1.1%)	10 (1.2%)
**Baseline proteinuria, g/d**		
Mean (SD)	2.8 (4.7)	2.9 (5.3)
Median (Q1-Q3)	1.2 (0.3-3.3)	1.5 (0.4-3.3)
**CCI**		
Mean (SD)	1.1 (1.6)	1.2 (1.5)
Median (Q1-Q3)	1.0 (0.0-1.0)	1.0 (0.0-2.0)
**Baseline CV events, n (%)**	165 (1.7)	10 (1.2)

*Note*: Because of rounding, percentages may not total 100.

Abbreviations: CCI, Charlson Comorbidity Index; CKD, chronic kidney disease; CV, cardiovascular; eGFR, estimated glomerular filtration rate; IgAN, immunoglobulin A nephropathy; KF, kidney failure; Q1-Q3, first quartile-third quartile.

a Excludes patients with baseline CKD stage 5.

The health care resource utilization/cost cohort consisted of 813 adult patients (aged ≥18 years) with linked claims data, having a mean age of 47.4 years and a median (Q1-Q3) 39.7 (21.9-59.8) months of post-index follow-up (Table 1). Sixty-two percent were male, and 74.9% were White. Median (Q1-Q3) proteinuria was 1.5 (0.4-3.3) g/d, and mean (standard deviation) proteinuria was 2.9 (5.3). Median (Q1-Q3) eGFR was 57.2 (29.2-86.6) mL/min/1.73 m^2^. Among patients with available eGFR data or a diagnosis code, 63.7% were in CKD stages 3-5 (Table 1). Both cardiovascular and kidney failure events were experienced by 1.2% of patients during the baseline period, and the median Charlson Comorbidity Index was mild (1.0 [0.0-1.0]).

### Prevalence

Among patients of all ages, the average annual estimated standardized prevalence of IgAN in the United States (2016-2020) was 329.0 per 1,000,000. An increasing trend in the prevalence of IgAN was observed over the study period (Table 2). Crude prevalence estimates by age and race are provided in Table 2.

**Table 2:** Standardized Prevalence Rate Per 1,000,000 US Population and Crude Prevalence by Age and Race

Year	Age Group	Crude Prevalence (95% CI)			Standardized Prevalence Per 1,000,000 US Population (95% CI)
		White	Black	Other	
2016	Total	269.6 (261.7-277.6)	110.0 (97.1-122.8)	414.7 (390.6-438.7)	270.0 (262.7-277.3)
	<18	144.0 (127.8-160.2)	30.7 (14.0-47.4)	89.9 (65.9-113.8)	
	18-44	294.6 (279.5-309.7)	122.1 (100.2-144.0)	476.0 (433.9-518.2)	
	45-64	404.4 (386.2-422.7)	167.8 (137.9-197.7)	678.1 (616.1-740.1)	
	>65	165.4 (153.7-177.0)	65.9 (42.3-89.5)	309.3 (257.5-361.0)	
2017	Total	312.5 (303.8-321.3)	130.6 (116.0-145.1)	499.2 (471.7-526.6)	313.4 (305.4-321.4)
	<18	150.6 (134.1-167.1)	47.1 (25.9-68.3)	88.8 (64.5-113.2)	
	18-44	339.8 (323.1-356.4)	139.5 (115.1-163.9)	580.1 (531.7-628.5)	
	45-64	471.8 (451.6-492.0)	199.1 (165.1-233.1)	824.8 (753.3-896.3)	
	>65	199.5 (186.2-212.7)	82.4 (54.7-110.1)	391.0 (329.6-452.3)	
2018	Total	349.5 (339.5-359.5)	141.0 (124.8-157.3)	564.2 (533.4-595.0)	345.2 (336.3-354.2)
	<18	138.3 (121.5-155.1)	37.2 (17.0-57.5)	114.6 (85.4-143.8)	
	18-44	376.7 (357.7-395.6)	151.3 (124.1-178.5)	615.7 (563.1-668.4)	
	45-64	529.6 (506.7-552.5)	220.4 (182.1-258.7)	926.4 (846.9-1,005.9)	
	>65	235.6 (219.8-251.4)	90.0 (58.3-121.7)	517.2 (442.0-592.3)	
2019	Total	337.9 (327.8-348.0)	133.2 (117.4-149.1)	559.3 (528.7-590.0)	333.7 (324.8-342.6)
	<18	126.5 (110.5-142.6)	36.6 (16.7-56.6)	90.6 (65.5-115.7)	
	18-44	358.8 (339.9-377.7)	146.1 (119.4-172.8)	566.8 (516.1-617.5)	
	45-64	509.5 (486.6-532.4)	191.1 (155.2-227.0)	1,003.4 (920.1-1,086.7)	
	>65	240.6 (223.8-257.3)	106.4 (71.1-141.6)	576.8 (495.4-658.2)	
2020	Total	403.1 (390.3-415.9)	154.2 (134.6-173.9)	587.4 (553.5-621.4)	382.5 (371.6-393.5)
	<18	141.4 (121.1-161.7)	34.6 (10.6-58.6)	115.1 (83.2-147.0)	
	18-44	411.4 (388.1-434.8)	160.9 (128.9-192.9)	562.5 (508.2-616.9)	
	45-64	598.8 (570.3-627.4)	227.7 (183.3-272.1)	1,064.6 (972.2-1,157.0)	
	>65	306.9 (284.8-329.0)	118.7 (76.2-161.2)	570.8 (487.2-654.5)	

Abbreviation: CI, confidence interval.

### Health Care Resource Utilization/Cost

#### By Proteinuria Level

**Figure 2:**
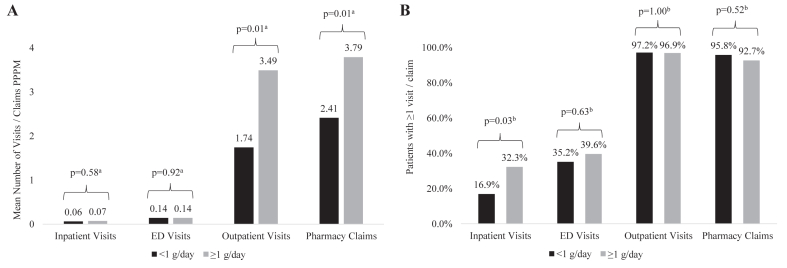
Unadjusted health care resource utilization by baseline proteinuria among Among Patients With IgAN, 2007-2020, n = 167. (A) Mean number of visits/claims PPPM. (B) Patients with ≥1 visit/claim. Abbreviations: ED, emergency department; IgAN, immunoglobulin A nephropathy; PPPM, per-patient-per-month. ^a^Linear regression. ^b^Fisher exact test.

Among patients with available baseline proteinuria data, after excluding those with baseline CKD stage 5/kidney failure (n = 167); 57.5% had high-risk proteinuria (≥1 g/d). High-risk proteinuria (≥1 g/d vs <1 g/d) in adult patients was associated with a higher mean PPPM number of outpatient visits (3.49 vs 1.74; *P* = 0.01) and pharmacy claims (3.79 vs 2.41; *P* = 0.01) (Fig 2A; Table S1). The proportion of patients with an inpatient stay was higher among patients with high-risk proteinuria (32.3% vs 16.9%; *P* = 0.03), whereas mean inpatient length of stay and mean number of inpatient visits PPPM were similar (Fig 2B; Table S1). Mean number of ED visits PPPM and the proportion of patients with an ED visit were not different for patients with high-risk baseline proteinuria.

**Figure 3:**
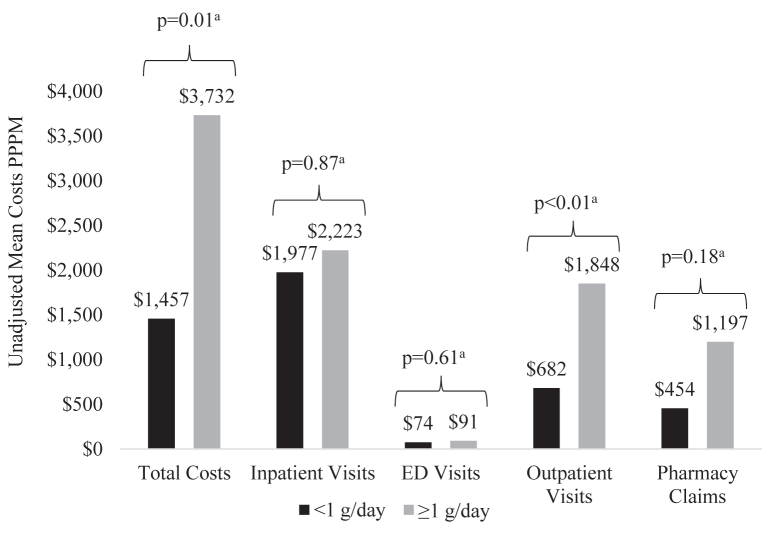
Unadjusted mean costs by baseline proteinuria level among Patients With IgAN, 2007-2020, n = 167. Abbreviations: ED, emergency department; IgAN, immunoglobulin A nephropathy; PPPM, per-patient-per-month. ^a^Linear regression.

Although there was a consistent trend to higher mean costs PPPM associated with high-risk proteinuria across individual elements of health care resource utilization, differences were only statistically significant for mean outpatient costs PPPM ($1,848 vs $682; *P* < 0.01). However, this trend resulted in a higher mean total costs PPPM for patients with high-risk proteinuria ($3,732 vs $1,457; *P* = 0.01) (Fig 3; Table S2).

#### By CKD Stage

**Figure 4:**
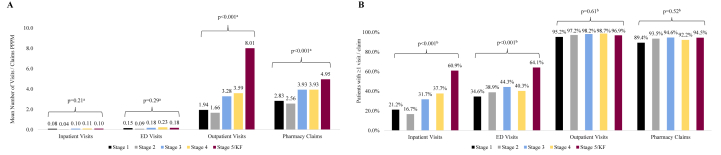
Unadjusted health care resource utilization by CKD stage Among Patients With IgAN, 2007-2020, n = 584. (A) Mean number of visits/claims PPPM. (B) Patients with ≥1 visit/claim. Abbreviations: CKD, chronic kidney disease; ED, emergency department; IgAN, immunoglobulin A nephropathy; KF, kidney failure; PPPM, per-patient-per-month. ^a^Linear regression. ^b^Fisher exact test.

**Figure 5:**
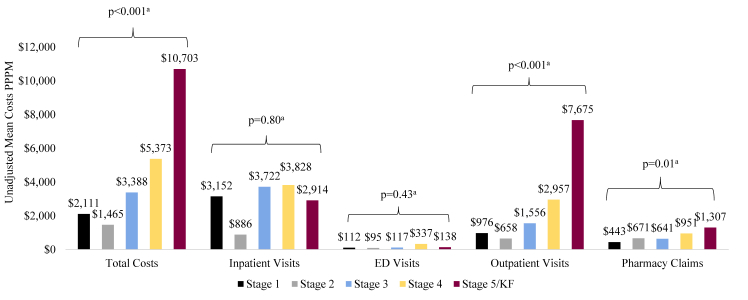
Unadjusted mean costs by CKD stage Among Patients With IgAN, 2007-2020, n = 584. Abbreviations: CKD, chronic kidney disease; ED, emergency department; IgAN, immunoglobulin A nephropathy; KF, kidney failure; PPPM, per-patient-per-month. ^a^Linear regression.

Higher CKD stage was associated with a trending higher mean number of outpatient visits and pharmacy claims PPPM (both *P* < 0.001; Fig 4B; Table S3). The proportion of patients with inpatient visits (*P* < 0.001) and ED visits (*P* < 0.001) increased with higher CKD stage. Mean total cost (PPPM; *P* < 0.001), mean/median costs associated with outpatient visits (*P* < 0.001), and mean (*P* = 0.01)/median (*P* < 0.001) costs associated with pharmacy claims also increased with higher CKD stage (Fig 5; Table S4).

## Discussion

An increasing prevalence of IgAN was observed between 2016 and 2020, which may indicate better disease awareness and diagnosis. High-risk proteinuria and advancing CKD stage from stage 1 to stage 5/kidney failure were both significantly associated with an increase in mean total cost PPPM, indicating that proteinuria and decline in kidney function are both associated with an increase in the economic burden of IgAN. To the best of our knowledge, this is the first study reporting the standardized prevalence of IgAN and utilizing a retrospective database to assess the impact of elevated proteinuria and higher CKD stages on health care resource utilization and costs of IgAN in the United States.

A previous systematic literature review^11^ identified 123 epidemiologic studies in IgAN with none reporting the prevalence of IgAN in the United States. However, the systematic review identified a study by Sim et al^18^ reporting an increasing trend of the incidence of IgAN in the United States, from 0.1 per 100,000 person-years in 2000 to 1.6 per 100,000 person-years in 2011, supporting the observed increase in prevalence of IgAN in the current study. Additionally, the authors conducted a meta-analysis on 5 US studies reporting IgAN rates in patients with kidney biopsy, reporting an estimated incidence of 1.29 per 100,000 persons based on 2019 US Census data.^11^

In our study, the mean number of outpatient visits and pharmacy claims increased significantly with higher CKD stage, whereas no differences were observed in the proportion of the patients with each type of visit because almost all patients had these visits. A higher number of outpatient visits and pharmacy claims is expected among patients with higher CKD stage because patients with more severe disease are likely to be interacting with their physician more often. No differences were observed in the mean number of inpatient and ED visits PPPM because they were relatively infrequent among the identified patients, but the proportion of patients with each type of visit increased significantly with higher CKD stage.

There was a consistent trend toward a higher PPPM health care resource utilization and costs associated with high-risk baseline proteinuria across the individual elements, with significant differences for mean outpatient visits and costs, and mean pharmacy claims, resulting in the higher observed total costs PPPM for patients with high-risk proteinuria. It was expected that the costs would be higher because these patients likely experience higher clinical burden. However, the nonsignificant results were likely due to small sample size among the baseline proteinuria cohorts caused by missing laboratory data and the small number of patients with IgAN. Patients with proteinuria <1 g/d are commonly perceived as being at lower risk and not considered for therapeutic intervention beyond renin-angiotensin system blockade. The unavailability of additional safe and effective treatments beyond renin-angiotensin-aldosterone system inhibitors for this group of patients will likely contribute to the lower costs and health care resource utilization compared with patients with high-risk proteinuria. It is important to note that although high-risk proteinuria was associated with higher health care resource utilization and costs in this study, proteinuria <1 g/d was also associated with a meaningful clinical and economic burden.

In the recent systematic literature review,^11^ only 3 studies were identified examining the economic burden of IgAN, including 2 retrospective database studies (1 in Canada and 1 in China) and 1 cost-effectiveness study in Japan.^11^, ^12^, ^13^, ^14^ The Canadian study assessed cost of immunosuppressive medications among patients with IgAN and did not analyze any outcomes comparable with the current study.^12^ The retrospective database analysis based in China reported overall health care resource utilization, including length of stay and emergency admissions; however, results were not stratified by proteinuria or CKD stage, limiting the ability to directly compare with our observations.^13^ The cost-effectiveness model that included patients with IgAN in Japan used previously reported costs associated with CKD stage.^14^ Increasing annual costs (2013 US dollars) from CKD stage 1 ($1,600) to stage 5 ($12,700) among Medicare patients were reported in the referenced publication; however, specific costs in patients with IgAN were not specified.^19^

The results reported in this study have several limitations, including those associated with using secondary databases as data sources for comparative analyses. For example, this study was limited to data in the Optum Market Clarity database and may not be representative of the broader US population, limiting generalizability. Additionally, missing data or errors are inherent in retrospective analyses, and those in detection of IgAN-related terms in patient records may introduce bias into the analyses including potential underestimation of US prevalence because there may be patients with IgAN who are unidentified. ICD codes were not used to identify patients because no specific codes are available for IgAN. Therefore, the presence of disease and symptoms terms were used to identify patients, and those with IgAN-related terms may have more severe disease because high-risk proteinuria is more likely to be present in the identified patients and bias results. The ability to identify kidney biopsies is limited in the database; thus, the patient population may have included patients with non–biopsy proven IgAN. Outliers may have inflated the mean baseline proteinuria estimates, causing a discrepancy with the median estimates. The patient population included about one-third of patients with unknown baseline CKD stage because of missing data required to calculate eGFR using the 2021 CKD-EPI equation, limiting the sample size used for the comparisons across CKD stage. Furthermore, among patients with available data, about 35% of patients had baseline CKD stage 4/5 because the analysis focuses on prevalent patients who are likely experiencing more severe disease. Cost data generated from this study are also associated with limitations with regard to the data source; Optum Market Clarity applies standard pricing algorithms to account for differences in pricing across health plans and provider contracts with resulting cost information designed to reflect allowed payments for all provider services across regions. Furthermore, the cost data provide a partial view of economic burden because out-of-pocket costs to patients were not included.

With the observed increasing prevalence of IgAN, and the increased health care resource utilization and costs among those with higher proteinuria and advancing CKD stage, treatments that reduce proteinuria and slow the rate of decline in kidney function have the potential to reduce the resource intensity and economic burden of IgAN.
