# Supplementary material for: Impact of Proteinuria and Kidney Function Decline on Health Care Costs and Resource Utilization in Adults With IgA Nephropathy in the United States: A Retrospective Analysis
Source: Kidney Med. 2023 Jun 25;5(9):100693. doi: 10.1016/j.xkme.2023.100693 (PMC10457441; doi:10.1016/j.xkme.2023.100693)
Supplement: Supplementary File (PDF) — Fig S1; Table S1-S4. [file mmc1.pdf]

Figure S1: Example Attrition Scenarios for SDS negation terms

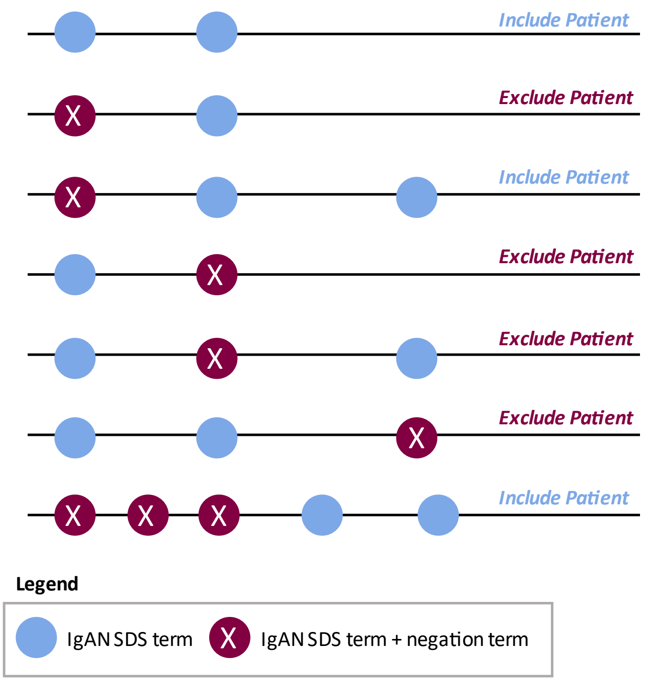

*Assuming eligible SDS terms are 30 days apart, within 180 days

Abbreviations: IgAN: immunoglobulin A nephropathy; SDS: signs, disease and symptoms

Table S1: Unadjusted Healthcare Resource Utilization by Baseline Proteinuria Level among IgAN patients, 2007 to 2020, n=167

Category	Baseline Proteinuria (g/day), n=167		
	**<1 g/day (n=71)**	**>=1 g/day (n=96)**	**P-value**
**Patients with ≥1 visit, n (%)**	71 (100.0)	94 (97.9)	0.51[a]
**Inpatient visits, n (%)**	12 (16.9)	31 (32.3)	0.03[a]
Visits PPPM			
Mean (SD)	0.06 (0.04)	0.07 (0.09)	0.58[b]
Median (Q1–Q3)	0.00 (0.00 – 0.10)	0.00 (0.00 – 0.10)	0.83[c]
Min (Max)	0.01 (0.14)	0.01 (0.44)	
LOS PPPM			
Mean (SD)	0.44 (0.50)	0.44 (0.68)	0.97[b]
Median (Q1–Q3)	0.20 (0.10 – 0.70)	0.20 (0.10 – 0.40)	0.91[c]
Min (Max)	0.01 (1.46)	0.02 (3.35)	
**ED visits, n (%)**	25 (35.2)	38 (39.6)	0.63[a]
Visits PPPM			
Mean (SD)	0.14 (0.31)	0.14 (0.19)	0.92[b]
Median (Q1–Q3)	0.10 (0.00 – 0.10)	0.10 (0.00 – 0.20)	0.15[c]
Min (Max)	0.01 (1.55)	0.01 (1.12)	
**Outpatient visits, n (%)**	69 (97.2)	93 (96.9)	>0.99[a]
Visits PPPM			
Mean (SD)	1.74 (1.62)	3.49 (4.85)	0.01[b]
Median (Q1–Q3)	1.20 (0.70 – 2.30)	2.00 (1.00 – 4.10)	<0.01[c]
Min (Max)	0.13 (10.46)	0.35 (33.48)	
**Pharmacy claims, n (%)**	68 (95.8)	89 (92.7)	0.52[a]
Claims PPPM			
Mean (SD)	2.41 (2.44)	3.79 (3.44)	0.01[b]
Median (Q1–Q3)	1.70 (0.80 – 3.20)	2.80 (1.20 – 5.60)	0.01[c]
Min (Max)	0.04 (13.49)	0.04 (15.48)	

Abbreviations: ED: emergency department; IgAN: immunoglobulin A nephropathy; LOS: length of stay; PPPM: per-patient-per-month; Q1–Q3: 1st quartile–3rd quartile; SD: standard deviation

[a]Fisher's exact test

[b]Linear regression

[c]Jonckheere–Terpstra test

Table S2: Unadjusted Costs by Baseline Proteinuria Level among IgAN patients, 2007 to 2020, n=167

**Category**	**Baseline Proteinuria (g/day), n=167**		
	**<1 g/day (n=71)**	**>=1 g/day (n=96)**	**P-value**
**Total costs PPPM**			
Mean (SD)	$1,457 ($2,467)	$3,732 ($6,624)	0.01[a]
Median (Q1–Q3)	$476 ($206 – $1,385)	$1,227 ($376 – $3,871)	<0.01[b]
Min (Max)	$2 ($12,439)	$25 ($38,247)	
**Inpatient visit costs PPPM**			
Mean (SD)	$1,977 ($2,242)	$2,223 ($4,987)	0.87[a]
Median (Q1–Q3)	$836 ($608 – $3,337)	$778 ($405 – $1,395)	0.45[b]
Min (Max)	$58 ($6,730)	$92 ($26,214)	
**ED visit costs PPPM**			
Mean (SD)	$74 ($107)	$91 ($134)	0.61[a]
Median (Q1–Q3)	$29 ($13 – $65)	$39 ($15 – $107)	0.59[b]
Min (Max)	$1 ($399)	$0 ($660)	
**Outpatient visit costs PPPM**			
Mean (SD)	$682 ($1,057)	$1,848 ($2,919)	<0.01[a]
Median (Q1–Q3)	$260 ($137 – $797)	$499 ($176 – $2,119)	0.01[b]
Min (Max)	$8 ($5,571)	$0 ($13,783)	
**Pharmacy claim costs PPPM**			
Mean (SD)	$454 ($1,455)	$1,197 ($4,410)	0.18[a]
Median (Q1–Q3)	$104 ($20 – $461)	$205 ($38 – $481)	0.14[b]
Min (Max)	$2 ($11,847)	$0 ($38,071)	

Abbreviations: ED: emergency department; IgAN: immunoglobulin A nephropathy; LOS: length of stay; PPPM: per-patient-per-month; Q1–Q3: 1st quartile–3rd quartile; SD: standard deviation

[a]Linear regression

[b]Jonckheere–Terpstra test

Table S3: Unadjusted Healthcare Resource Utilization by CKD Stage among IgAN patients, 2007 to 2020, n=584

**Category**	**Baseline CKD stage (n=584)**					
	**Stage 1 (n=104)**	**Stage 2 (n=108)**	**Stage 3 (n=167)**	**Stage 4 (n=77)**	**Stage5/KF (n=128)**	**P-value**
**Patients with ≥1 visit, n (%)**	**100 (96.2)**	**107 (99.1)**	**166 (99.4)**	**77 (100.0)**	**126 (98.4)**	**0.21[a]**
**Inpatient visits, n (%)**	22 (21.2)	18 (16.7)	53 (31.7)	29 (37.7)	78 (60.9)	<0.001[a]
Visits PPPM						
Mean (SD)	0.08 (0.08)	0.04 (0.03)	0.10 (0.11)	0.11 (0.23)	0.10 (0.13)	0.21[b]
Median (Q1–Q3)	0.00 (0.00 – 0.10)	0.00 (0.00 – 0.00)	0.10 (0.00 – 0.10)	0.00 (0.00 - 0.10)	0.10 (0.00 - 0.10)	0.36[c]
Min (Max)	0.01 (0.28)	0.01 (0.11)	0.01 (0.59)	0.01 (1.20)	0.01 (0.68)	
LOS PPPM						
Mean (SD)	0.77 (1.31)	0.16 (0.12)	0.91 (1.63)	0.78 (2.28)	0.67 (1.38)	0.94[b]
Median (Q1–Q3)	0.20 (0.10 – 0.50)	0.10 (0.10 – 0.20)	0.20 (0.20 – 1.00)	0.20 (0.10 - 0.50)	0.20 (0.10 - 0.50)	0.70[c]
Min (Max)	0.03 (4.98)	0.03 (0.49)	0.01 (8.33)	0.02 (12.42)	0.01 (8.37)	
**ED visits, n (%)**	36 (34.6)	42 (38.9)	74 (44.3)	31 (40.3)	82 (64.1)	<0.001[a]
Visits PPPM						
Mean (SD)	0.15 (0.19)	0.09 (0.14)	0.18 (0.28)	0.23 (0.71)	0.18 (0.26)	0.29[b]
Median (Q1–Q3)	0.10 (0.00 – 0.20)	0.00 (0.00 – 0.10)	0.10 (0.00 – 0.10)	0.10 (0.00 - 0.10)	0.10 (0.00 - 0.20)	0.04[c]
Min (Max)	0.01 (0.90)	0.01 (0.56)	0.01 (1.55)	0.01 (4.04)	0.02 (1.22)	
**Outpatient visits, n (%)**	99 (95.2)	105 (97.2)	164 (98.2)	76 (98.7)	124 (96.9)	0.61[a]
Visits PPPM						
Mean (SD)	1.94 (1.88)	1.66 (1.65)	3.28 (4.54)	3.59 (3.81)	8.01 (7.29)	<0.001[b]
Median (Q1–Q3)	1.30 (0.70 – 2.50)	1.30 (0.70 – 2.00)	1.90 (1.00 – 3.70)	2.80 (1.10 – 4.40)	5.40 (2.70 – 10.40)	<0.001[c]
Min (Max)	0.04 (9.44)	0.07 (13.61)	0.02 (33.48)	0.11 (21.55)	0.27 (31.93)	
**Pharmacy claims, n (%)**	93 (89.4)	101 (93.5)	158 (94.6)	71 (92.2)	121 (94.5)	0.52[a]
Claims PPPM						
Mean (SD)	2.83 (3.09)	2.56 (2.85)	3.93 (3.22)	3.93 (3.64)	4.95 (3.41)	<0.001[b]
Median (Q1–Q3)	1.50 (0.80 – 4.00)	1.60 (0.09 – 3.40)	3.10 (1.40 – 6.00)	3.30 (1.40 - 5.20)	4.50 (2.40 - 7.10)	<0.001[c]
Min (Max)	0.05 (13.49)	0.03 (20.95)	0.01 (15.48)	0.04 (21.92)	0.02 (17.38)	

Abbreviations: CKD: chronic kidney disease; ED: emergency department; IgAN: immunoglobulin A nephropathy; KF: kidney failure; LOS: length of stay; PPPM: per-patient-per-month; Q1–Q3: 1st quartile–3rd quartile; SD: standard deviation

[a]Fisher's exact test

[b]Linear regression
[c]Jonckheere–Terpstra test

Table S4: Unadjusted Costs by CKD Stage among IgAN patients, 2007 to 2020, n=584

**Category**	**Baseline CKD stage (n=584)**					
	**Stage 1 (n=104)**	**Stage 2 (n=108)**	**Stage 3 (n=167)**	**Stage 4 (n=77)**	**Stage5/KF (n=128)**	**P-value**
**Total Costs PPPM**						
Mean (SD)	$2,111 ($3,992)	$1,465 ($3,769)	$3,388 ($5,610)	$5,373 ($9,425)	$10,703 ($16,559)	<0.001[a]
Median (Q1 - Q3)	$688 ($173 – $2,065)	$695 ($262 – $1,650)	$1,224 ($367 – $3,622)	$1,717 ($633 – ($5,668)	$5,853 ($2,922 – $13,022)	<0.001[b]
Min (Max)	$0 ($26,734)	$7 ($38,247)	$2 ($36,092)	$9 ($68,060)	$28 ($153,700)	
**Inpatient visit costs PPPM**						
Mean (SD)	$3,152 ($5,785)	$886 ($597)	$3,722 ($6,461)	$3,828 ($8,869)	$2,914 ($5,264)	0.80[a]
Median (Q1 - Q3)	$833 ($544 - $2,143)	$723 ($405 - $1,244)	$1,271 ($620 - $4,799)	$1,032 ($532 - $2,369)	$1,223 ($574 - $2,988)	0.32[b]
Min (Max)	$66 ($21,718)	$186 ($2,119)	$58 ($31,249)	$92 ($46,936)	$61 ($32,999)	
**ED visit costs PPPM**						
Mean (SD)	$112 ($171)	$95 ($271)	$117 ($209)	$337 ($1,376)	$138 ($238)	0.43[a]
Median (Q1 - Q3)	$41 ($16 - $138)	$13 ($6 - $59)	$39 ($18 - $104)	$35 ($7 - $142)	$41 ($16 - $99)	0.22[b]
Min (Max)	$0 ($794)	$0 ($1,353)	$0 ($1,142)	$0 ($7,719)	$0 ($943)	
**Outpatient visit costs PPPM**						
Mean (SD)	$976 ($1,500)	$658 ($775)	$1,556 ($2,435)	$2,957 ($3,765)	$7,675 ($15,460)	<0.001[a]
Median (Q1 - Q3)	$455 ($110 - $1,037)	$356 ($155 - $841)	$536 ($171 - $1,984)	$1,327 ($264 - $4,180)	$3,250 ($1,276 - $8,706)	<0.001[b]
Min (Max)	$0 ($7,714)	$0 ($4,007)	$0 ($13,083)	$21 ($14,048)	$50 ($151,893)	
**Pharmacy claim costs PPPM**						
Mean (SD)	$443 ($1,118)	$671 ($3,794)	$641 ($1,461)	$951 ($1,931)	$1,307 ($3,054)	0.01[a]
Median (Q1 - Q3)	$65 ($15 - $398)	$104 ($22 - $281)	$201 ($47 - $616)	$306 ($105 - $884)	$670 ($243 - $1,456)	<0.001[b]
Min (Max)	$1 ($7,168)	$1 ($38,071)	$0 ($11,847)	$1 ($13,473)	$0.4 ($28,710)	

Abbreviations: CKD: chronic kidney disease; ED: emergency department; IgAN: immunoglobulin A nephropathy; Q1–Q3: 1st quartile–3rd quartile; KF, kidney failure; LOS: length of stay; PPPM: per-patient-per-month; SD: standard deviation

[a]Linear regression

[b]Jonckheere–Terpstra test
